# Effect of a Probiotic Combination in an Experimental Mouse Model and Clinical Patients With Chronic Kidney Disease: A Pilot Study

**DOI:** 10.3389/fnut.2021.661794

**Published:** 2021-05-31

**Authors:** I-Kuan Wang, Tzung-Hai Yen, Pei-Shan Hsieh, Hsieh-Hsun Ho, Yi-Wei Kuo, Yen-Yu Huang, Yu-Lun Kuo, Chi-Yuan Li, Hung-Chih Lin, Jiu-Yao Wang

**Affiliations:** ^1^Division of Nephrology, China Medical University Hospital, Taichung, Taiwan; ^2^Department of Internal Medicine, China Medical University College of Medicine, Taichung, Taiwan; ^3^Division of Nephrology, Chang Gung Memorial Hospital, Taipei, Taiwan; ^4^Department of Internal Medicine, Chang Gung University College of Medicine, Taoyuan, Taiwan; ^5^Glac Biotech Co., Ltd., Tainan, Taiwan; ^6^Biotools Co., Ltd., New Taipei City, Taiwan; ^7^Department of Anesthesiology, China Medical University Hospital, Taichung, Taiwan; ^8^Graduate Institute of Biological Science, China Medical University College of Medicine, Taichung, Taiwan; ^9^Division of Neonatology, China Medical University Children's Hospital, China Medical University, Taichung, Taiwan; ^10^School of Chinese Medicine, College of Chinese Medicine, China Medical University, Taichung, Taiwan; ^11^Asia University Hospital, Asia University, Taichung, Taiwan; ^12^Department of Pediatrics, National Cheng Kung University Hospital, Tainan, Taiwan; ^13^Children's Hospital, China Medical University, Taichung, Taiwan

**Keywords:** probiotics, chronic kidney disease, microbiota, microorganisms, renal function

## Abstract

The aim of the present study was to evaluate whether probiotic administration could slow declining renal function. C57BL/6 mice (6–8 weeks of age, male) were fed a diet supplemented with adenine to induce chronic kidney disease (CKD). The experimental groups were additionally supplemented with 10^9^ colony-forming units (CFU)/day (high-dose) and 10^7^ CFU/day (low-dose) probiotics containing *Lactobacillus acidophilus* (TYCA06), *Bifidobacterium longum* subspecies *infantis* (BLI-02), and *B. bifidum* (VDD088). Renal function and histology were examined. Patients with stage 3–5 CKD and not on dialysis were recruited from July 2017 to January 2019. Two capsules of probiotics containing 2.5 × 10^9^ CFU with the same composition were administered twice daily for 6 months. The decline in the estimated glomerular filtration rate (eGFR) was measured before and after the intervention. In addition, changes in the serum endotoxin and cytokine levels, gastrointestinal symptom scores, and the stool microbiota were measured. Probiotics could attenuate renal fibrosis and improve renal function in CKD mice. Thirty-eight patients completed the 6-month study. The mean baseline eGFR was 30.16 ± 16.52 ml/min/1.73 m^2^. The rate of decline in the eGFR was significantly slower, from −0.54 (−0.18, −0.91) to 0.00 (0.48, −0.36) ml/min/1.73 m^2^/month (*P* = 0.001) after 6 months of treatment. The serum levels of TNF-α, IL-6, IL-18, and endotoxin were significantly decreased after probiotic administration. Borborygmus and flatulence scores, as well as stool formation improved significantly. The abundance of *B. bifidum* and *B. breve* in the stool microbiota increased significantly. In conclusion, a combination of probiotics might attenuate renal function deterioration in CKD mice and human patients.

## Introduction

Chronic kidney disease (CKD), an important global issue, affects ~10% of the adult population and has a negative economic impact on healthcare systems (1). Current strategies for CKD management include blood pressure control, glycemic control, and low protein and sodium intake (2). There is no effective therapy for this disease to date. Innovative approaches to manage and control, and even therapy for the disease are urgently needed.

The microbiota is a collection of microorganisms living in a commensal relationship with their host. In humans, the gastrointestinal tract contains up to 100 trillion micro-organisms, among which there are more than 1,000 different species (3). These microbes can modulate the immune system, protect against pathogen invasion, and regulate metabolism (4). The intestinal microbiota are altered, both quantitatively and qualitatively, in patients with CKD (5, 6). A previous study analyzed the gut microbiota using real-time PCR and found that *Bifidobacterium* species, *B. catenulatum, B. longum, B. bifidum, Lactiplantibacillus plantarum* (formerly *Lactobacillus plantarum*), and *Lacticaseibacillus paracasei* (formerly *Lactobacillus paracasei*) were detected at lower rates in patients on peritoneal dialysis (PD) compared with healthy controls. Disturbance of the normal gut microbiota contributed to chronic inflammation in patients with CKD through the generation and influx of endotoxin, microbial fragments, and other toxic and pro-inflammatory products (7). Systemic inflammation leads to progressive renal fibrosis and associated complications, such as cardiovascular disease, cachexia, and anemia (8, 9).

The International Scientific Association for Probiotics and Prebiotics (ISAPP) consensus defines probiotics as “live microorganisms which when administered in adequate amounts confer a health benefit on the host” (10). They could benefit the host by inhibiting the growth and invasion of pathogens, improving intestinal barrier function, and regulating the immune system (11–14). Interventional studies investigating the efficacy of probiotics in patients with CKD are limited and have produced conflicting results (15, 16).

The primary endpoint of this study was whether probiotics could attenuate the progression of CKD in mice as well as in humans. The secondary endpoints of this study were the effects of probiotics on the serum levels of endotoxin and pro-inflammatory cytokines, gastrointestinal symptoms, and the stool microbiota in patients with CKD.

## Materials and Methods

### Food-Grade Probiotics

Food-grade probiotics were selected by performing *in vitro* studies. *L. acidophilus, B. longum* subspecies *infantis*, and *B. bifidum* were defined as edible probiotic strains with Generally Recognized as Safe (GRAS) status. This study examined the effects of these probiotics in combination in both uremic mice and patients with CKD.

### Indole Assay

Probiotics (1 × 10^9^ CFU) were cultured in tubes containing 5 ml de Man, Rogosa, and Sharpe (MRS) agar medium at 37°C for 18 h. *B. animalis* subspecies *lactis* BB-12 (Chr. Hansen, Hoersholm, Denmark), *B. bifidum* VDD088 isolated from health infant gut (Glac Biotech Co., Ltd., Tainan, Taiwan), and *B. longum* subspecies *infantis* BLI-02 isolated from breast milk (Glac Biotech Co., Ltd., Tainan, Taiwan) were incubated under anaerobic conditions. *L. rhamnosus* (formerly *Lactobacillus rhamnosus*) GG from the American Type Culture Collection (ATCC) 53103, and *L. acidophilus* TYCA06 isolated from healthy human gut (Glac Biotech Co., Ltd., Tainan, Taiwan) were incubated under facultative anaerobic conditions. *Escherichia coli* from the ATCC 8739 were incubated under aerobic conditions. Tryptone water was used to detect indole production from microorganisms. Indole (1 mM) was used as a positive control. After incubation, media containing probiotics were centrifuged at 3,000 revolutions per minute (rpm) for 5 min, and then supernatants were discarded and tryptone water was added to each culture tube. Probiotics mixed with tryptone water were re-incubated at 37°C for 48 h and were centrifuged at 3,000 rpm for 5 min. After centrifugation, the supernatant was transferred to a sterilized transparent glass tube and then 0.2 ml Kovac's reagent was added for 5 min. Finally, optical density (OD) 540 nm was used to detect indole levels.

### Indole Assay: Co-culture of Probiotics and *E. coli*

*B. animalis* subspecies *lactis* (BB-12), *B. bifidum* (VDD088), *B. longum* subspecies *infantis* (BLI-02), *L. rhamnosus* (LGG), *L. acidophilus* (TYCA06), *L. plantarum* L-29 (Glac Biotech Co., Ltd., Tainan, Taiwan), all at 1 × 10^9^ CFU, and a mixed group containing a 1:1:1 ratio of *L. acidophilus* (TYCA06), *B. longum* subspecies *infantis* (BLI-02), and *B. bifidum* (VDD088) were co-cultured with *E. coli* (1 × 10^6^ CFU) in tubes containing 5 ml of tryptone water under facultative anaerobic conditions at 37°C for 16 h. After incubation, the co-cultured probiotics mixed with tryptone water were centrifuged at 3,000 rpm for 5 min, and then the supernatants were transferred to sterilized, transparent glass tubes. Finally, 0.2 ml Kovac's reagent was added for 5 min and OD 540 nm was used to detect indole levels. The indole level of the *E. coli* group was as regarded as a control.

### Antimicrobial Ability of Probiotics

*L. acidophilus* (TYCA06), *B. longum* subspecies *infantis* (BLI-02), *B. bifidum* (VDD088), *B. animalis* subspecies *lactis* (BB-12), *L. rhamnosus* (LGG), and *L. plantarum* (L-29) were inoculated on MRS agar plates in the form of 2-cm-wide strips and cultured at 37°C for 48 h. The plates were then overlaid and solidified with tryptone soy (TS) agar and then *E. coli* was streaked with a cotton swab over the TS agar on the plate containing the above probiotic cultures. The plates were re-incubated at 37°C for 48 h, and the inhibition zones for *E. coli* were measured and expressed as follows: (–), no detectable zone of inhibition; (+), 0–2 cm growth inhibition; (++), 2–3 cm growth inhibition; (+++), 3–4 cm growth inhibition; (++++), 4–5 cm growth inhibition, and (+++++), 5–8.5 cm growth inhibition.

### Animal Studies

C57BL/6 mice (6–8 weeks of age, male) were investigated in National Cheng Kung University and Hospital, Tainan, Taiwan. All the animals were housed in groups of 5–6 in sterilized cages fitted with filter cage tops and fed with sterilized food and water. Animals were housed in specific-pathogen-free animal facilities under controlled temperature (22 ± 2°C) and humidity (62 ± 5%) conditions with a 12-h light–dark cycle, were given standard chow, and had access to water *ad libitum*. Animal experiments and protocols were in compliance with the NIH Guide for the Care and Use of Laboratory Animals. All animal experiments were performed according to protocols approved by the Animal Ethics Committee and the Institutional Animal Care and Use Committee (IACUC No. 106162), College of Medicine, National Cheng Kung University, Tainan, Taiwan.

### Probiotic Strains and Cultivation

Active and dry *L. acidophilus* (TYCA06), *B. longum* subspecies *infantis* (BLI-02), and *B. bifidum* (VDD088) were administered in a ratio of 1:1:1. The concentration of live bacteria was 10^11^ CFU/g after multi-strain product preparation. For experimental use, *Lactobacillus* and *Bifidobacterium* species were cultured with MRS broth containing 0.05% cysteine. Probiotics were incubated under anaerobic conditions at 37°C for 20 h and further dried by lyophilization. Probiotic viability was determined using a CFU assay.

### Murine CKD Model and Probiotic Treatment

The mice were divided at random into four groups (five to six mice/group). Adenine was purchased from Sigma-Aldrich, Inc. (Saint Louis, MO, USA). To induce CKD, the mice were fed daily with sterilized food containing 0.2% adenine (Ad-mice) for 42 days. Mice in the control group were fed with sterilized food only. The other three groups were Ad-mice with additional daily supplementation by oral gavage with a 10^9^ CFU/day (high-dose), 10^7^ CFU/day (low-dose) probiotic combination, and phosphate-buffered saline (PBS) only. The food and water intake of each group were remained unchanged and were monitored weekly. All animals were sacrificed on day 42. The kidney tissues were collected and analyzed. Blood samples in each group were collected 1 day before sacrifice for further biochemical analysis.

### Physical Property Analysis and Serum Biochemical Analysis

Mouse blood pressure was measured on day 41 using Visitech BP-2000 System (Visitech Systems, Inc., USA), a non-invasive continuous blood pressure monitor. The mobility of the mice was restricted. The machine indicators were fixed on their tails to measure blood pressure. Prior to blood pressure measurement, the animals were allowed to adapt to the stress derived from blood-pressure detection over 3 days of continuous monitoring. Body and kidney weight were analyzed on day 42. For detection of blood urea nitrogen (BUN) and creatinine, blood samples were placed on test strips. The strips were analyzed by FUJI DRI-CHEM 4000i (Fujifilm Corporation, Japan).

### Histology

The kidneys were fixed in 10% neutral formalin for 24 h. The kidney tissues were further dehydrated in a graded series of ethanol and eventually embedded in paraffin blocks. Sections of the renal cortex, renal medulla, and renal pelvis were made and stained with H&E for observation of immune cell infiltration. Masson's trichrome (MT) staining was performed for the detection of fibrosis in the kidneys.

### Clinical Trial Design and Ethics Statements

The clinical trial was conducted in compliance with the Declaration of Helsinki. Informed consent was obtained from all participants. The trial protocol was approved by the Institutional Review Board and registered in clinical trials (NCT03228563). Patients with CKD were recruited at China Medical University Hospital, Taichung, Taiwan from July 2017 to January 2019. Inclusion criteria included stages 3–5 CKD not on dialysis, at least 20 years of age, and regular follow-up for at least 6 months prior to enrollment. The exclusion criteria included pregnancy, being on immunosuppressive therapy, active infectious conditions, acute kidney injury, or the consumption of other forms of probiotics or antibiotics within 30 days prior to enrollment.

### Study Design

Patients with CKD were supplemented with two capsules containing 2.5 × 10^9^ CFU *L. acidophilus* (TYCA06), *B. longum* subspecies *infantis* (BLI-02), and *B. bifidum* (VDD088) in a ratio of 1:1:1 after meals in the morning and evening daily for 6 months. The participants had been educated by dietitians before the study to consume a low-protein diet and were advised not to change their dietary habits or lifestyles during the trial period. The estimated glomerular filtration rate (eGFR) and serum levels of endotoxin and inflammatory cytokines (TNF-α, IL-6, and IL-18) were examined before as well as 3 and 6 months after the probiotic intervention. In addition, stool samples were collected from the participants, and next-generation sequencing (NGS) was performed to evaluate the change in the gut microbiota after the intervention. Gastrointestinal symptoms were also studied *via* a questionnaire.

### Assessment of eGFR, Endotoxin, and Proinflammatory Cytokines

Blood samples were collected after 8 h of fasting. After centrifugation, the serum was obtained and frozen at −80°C. The eGFR values were calculated by the IDMS-MDRD method [175 × Cr^−1.154^ × age^−0.203^ × (0.742, if female) × (1.212, if African American)] (17). The rate of decline in the eGFR was compared between baseline and after the intervention. The serum levels of TNF-α, IL-6, and IL-18 were also measured prior to and after probiotic treatment using ELISA methods (Thermo Fisher Scientific, USA). Serum endotoxin levels were quantified by Chromogenic Limulus Amebocyte Lysate test (Thermo Fisher Scientific, USA). All samples were measured in duplicate.

### Evaluation of Gastrointestinal Symptoms

Gastrointestinal symptoms were evaluated by a study nurse using a questionnaire at baseline as well as 3 months and 6 months after the intervention (18). The questionnaire included the stool form (1 = very hard [small hard lumps], 2 = hard stool [hard sausage shape], 3 = normal stool [sausage or banana shape], 4 = soft stool, 5 = muddy stool, 6 = watery stool), ease of defecation (1 = difficult, 2 = easy, 3 = very easy), and abdominal symptoms [frequency of upper abdominal pain, lower abdominal pain, borborygmus, and flatulence (1 = frequent, 2 = occasional, 3 = almost never)]. The average scores before and after the intervention were analyzed.

### Stool Collection and the NGS Assay

Stools from the 36 participants were collected before and after probiotic treatment and immediately stored at −80°C. For the NGS assay, the DNA of fecal samples (about 100 mg) was extracted using the Quick-DNA™ Fungal/Bacterial Miniprep Kit (ZYMO Research, USA). DNA concentration and purity were monitored on 1% agarose gels. Based on the concentration, the DNA was diluted to 1 ng/μl using sterile water. NGS assays were performed. In brief, the amplicon DNA segments (16S rRNA, 16S V3-V4) were amplified by PCR (Phusion® High-Fidelity PCR Master Mix, New England Biolabs, USA) using specific primers. The DNA products between 400 and 450 base pairs were purified with the Qiagen Gel Extraction Kit (Qiagen, Germany). Sequencing libraries were generated using the TruSeq® DNA PCR-Free sample preparation kit (Illumina, USA), and index codes were provided. Library quality was assessed using the Qubit@ 2.0 Fluorometer (Thermo Fisher Scientific, USA) and Agilent Bioanalyzer 2100 system (Agilent Technologies, Inc., USA). The library was sequenced on an Illumina HiSeq 2500 platform (Illumina, USA). For reads assembly, the entire paired-end reads were assembled using FLASH v.1.2.11. As a quality control, low-quality (Q < 20) reads were discarded in the QIIME 1.9.1 pipeline. Amplicons with chimeric sequences were removed using UCHIME to obtain the effective tags. Clustering operational taxonomic units (OTU) with a 97% similarity sequence were identified by UPARSE function in the USEARCH v.7 pipeline. The Silva Database v.132 was used based on the RDP classifier (Version 2.2) algorithm to annotate taxonomic information. Sequences that appearing once (singletons) or measured in only one sample were filtered out. The complexity of species for each sample was analyzed in alpha diversity analysis and the differences between microbial communities or samples were evaluated in beta diversity analysis. Weighted and unweighted UniFrac parameters in beta-diversity were calculated by QIIME pipeline. After calculating the beta diversity and generating principal coordinates, multidimensional visualization of Principal Coordinate Analysis (PCoA) was presented by using the FactoMineR package and ggplot2 package in R software (v.3.3.1).

### Statistical Analysis

Continuous variables were expressed as mean ± SD or median and 25th−75th percentile as appropriate. A Kruskal–Wallis test or Wilcoxon signed-rank test was applied to examine the differences between continuous variables with SPSS, Version 12.0 (SPSS Inc., Chicago, IL, United States). For statistical analysis of NGS results, various taxonomic levels among groups were detected using differential abundance analysis with a zero-inflated Gaussian log-normal model as implemented in the Gaussian function of the Bioconductor metagenomeSeq package (19). The significance of differences in the relative abundance distribution at each taxonomic level among groups was calculated by a permutation test. Differences between probiotic species were analyzed as previously described (19, 20). *P* < 0.05 was the criterion for significance.

## Results

### Selecting Potential Probiotics by *in vitro* Indole Assay for the Treatment of CKD

**Figure 1:**
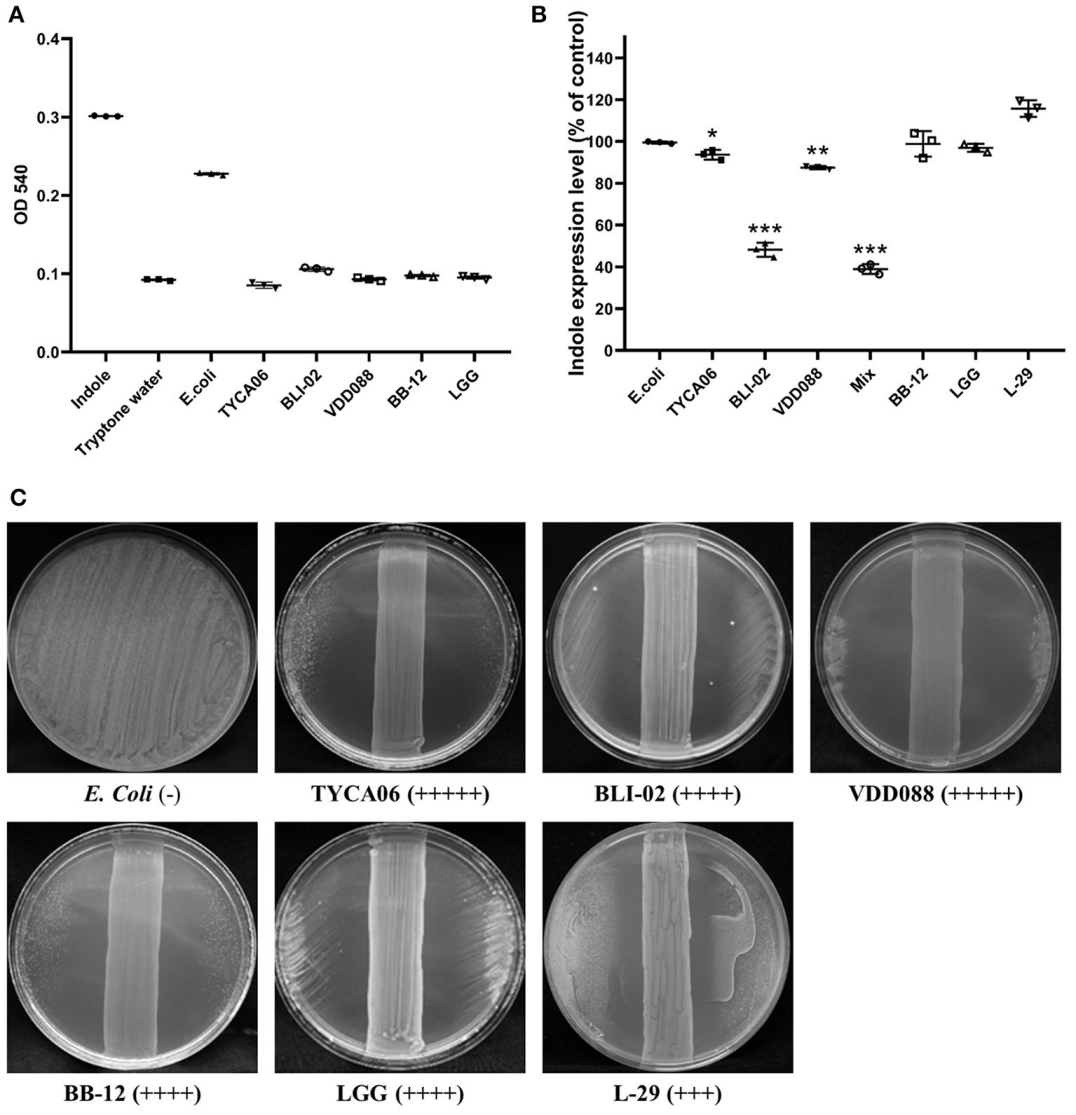
Effect of different probiotic strains on indole production. **(A)** The indole levels produced by individual probiotic strains were measured. Indole (1 mM) was added as a positive control, while tryptone water was added as a negative control. **(B)** Indole levels in co-cultures of *E. coli* and probiotics. The *E. coli* group was regarded as a control. Other bacterial groups [*Lactobacillus acidophilus* (TYCA06)*, Bifidobacterium longum* subspecies *infantis* (BLI-02), *Bifidobacterium bifidum* (VDD088), *Bifidobacterium animalis* subspecies *lactis* (BB-12), *L. rhamnosus* GG (LGG), *and L. plantarum* (L-29)] were co-cultured with *E. coli*. The mixed group indicates the co-cultures of *Lactobacillus acidophilus* (TYCA06), *Bifidobacterium longum* subspecies *infantis* (BLI-02), *Bifidobacterium bifidum* (VDD088), and *E. coli*. *P < 0.05, **P < 0.01, ***P < 0.001, as compared with the *E. coli* group. **(C)** Antimicrobial ability of different probiotic strains. (–), no detectable zone of inhibition for *E. coli*; (+++), 3–4 cm inhibition; (++++), 4–5 cm inhibition; (+++++), 5–8.5 cm inhibition. Different probiotic strains included *Lactobacillus acidophilus* TYCA06 (TYCA06), *Bifidobacterium longum* subspecies *infantis* BLI-02 (BLI-02), *Bifidobacterium bifidum* VDD088 (VDD088), *Bifidobacterium animalis* subspecies *lactis* BB-12 (BB-12), *L. rhamnosus* GG (LGG), and *L. plantarum* L-29 (L-29).

Indole is generated and absorbed by intestinal probiotics and reaches liver cells through the blood circulation. In liver cells, indole is metabolized and converted into cytotoxic indoxyl sulfate (21). Under normal circumstances, indoxyl sulfate can be cleared effectively by the kidneys. However, people with poor renal function cannot eliminate indoxyl sulfate. A large amount of indoxyl sulfate will accumulate and then circulate from the blood to the kidneys or other organs, causing cytotoxicity in those organs. Therefore, our probiotic combination for CKD was screened and based on the results of the indole tests, its potential anti-microbial activity against pathogenic strains such as *E. coli* was determined. Our screening results showed that there was almost no indole production in the culture media of *L. acidophilus* (TYCA06), *B. longum* subspecies *infantis* (BLI-02), or *B. bifidum* (VDD088) (Figure 1A).

*E. coli* is an indole-producing bacterium; therefore, indole levels were further measured in probiotic and *E. coli* co-cultures. Indole production in the co-culture media treated with *L. acidophilus* (TYCA06), *B. longum* subspecies *infantis* (BLI-02), *B. bifidum* (VDD088), and the combination of *Lactobacillus acidophilus* (TYCA06), *Bifidobacterium longum* subspecies *infantis* (BLI-02), and *Bifidobacterium bifidum* (VDD088), was effectively reduced to 92.58% (*P* = 0.042), 46.64% (*P* = 0.000), 87.35% (*P* = 0.006), and 37.85% (*P* = 0.000), as compared with the *E. coli* group, respectively (Figure 1B).

The results of an anti-microbial assay with co-cultures of probiotics and *E. coli* on an agar plate revealed that *L. acidophilus* (TYCA06), *B. longum* subspecies *infantis* (BLI-02), and *B. bifidum* (VDD088) effectively restricted the growth of *E. coli*. LGG, L-29, or BB-12 also had inhibitory effects on *E. coli* growth (Figure 1C). Taken together, the authors suggested that co-cultures of probiotics and *E. coli* could decrease the production of indole through the inhibition of *E. coli* growth. Based on these results, the combination of three probiotic strains, *L. acidophilus* (TYCA06), *B. longum* subspecies *infantis* (BLI-02), and *B. bifidum* (VDD08), were selected for follow-up animal and clinical studies.

### Probiotics Attenuated Clinical Symptoms and Pathological Findings of CKD in an Ad-Induced Mice With CKD

**Figure 2:**
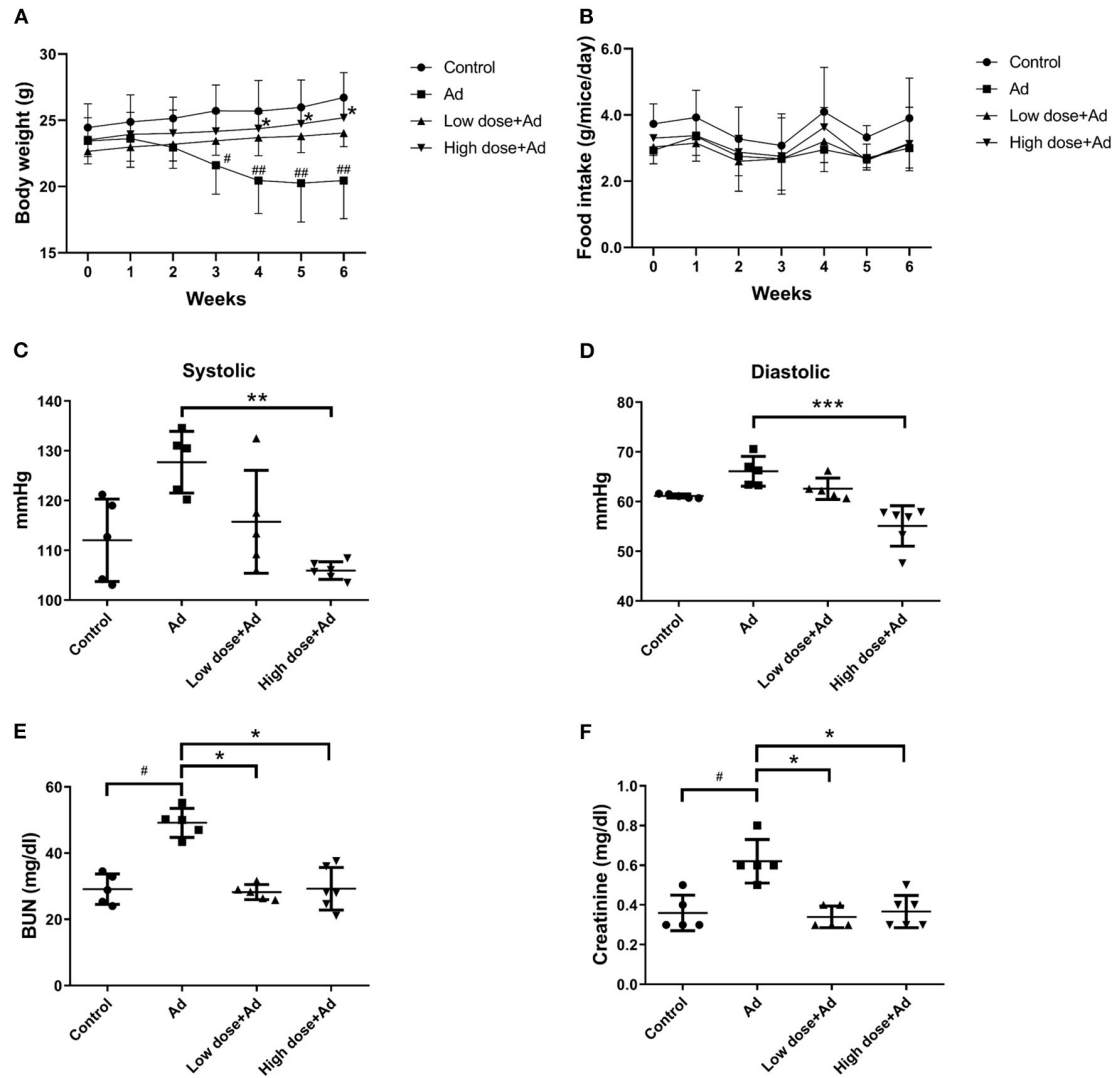
Physical properties and biochemical analyses of mice with adenine (Ad)-induced chronic kidney disease (CKD) after 6 weeks of probiotic treatment. **(A)** body weight measurement, **(B)** food intake measurement, **(C,D)** systolic and diastolic blood pressure measurement, **(E)** serum blood urea nitrogen (BUN) levels, **(F)** serum creatinine levels. Mice without any treatments were used as experimental controls. The graphs represent the mean ± SD obtained from five to six mice per group. Statistical analysis was performed by Kruskal–Wallis test. ^#^,*P < 0.05; ^*##*^, **P < 0.01; ***P < 0.001. Asterisk means the significant difference between Ad and high dose+Ad group, or Ad and low dose+Ad group; pound means a significant difference between control and Ad group.

Mice supplemented with adenine (Ad-mice) for 6 weeks showed reduced body weight (Figure 2A), but the food intake was not changed significantly (Figure 2B). Typical pathophysiological symptoms of CKD such as systolic and diastolic blood pressure were also elevated (Figures 2C,D), when compared with control mice. There was a significant increase in body weight (*P* = 0.038) and a decrease in systolic (*P* = 0.007) and diastolic (*P* = 0.000) pressure in an Ad-mice administered a high dose of probiotics. From biochemical studies, Ad-mice had significantly higher serum BUN (*P* = 0.048) and creatinine (*P* = 0.038) levels than controls (Figures 2E,F), and Ad-mice treated with both low and high doses of probiotics had significantly reduced serum levels of BUN and creatinine, compared with Ad-mice without treatment (low dose of probiotics: *P* = 0.048 and *P* = 0.042, respectively; high dose of probiotics: *P* = 0.027 and *P* = 0.019, respectively).

**Figure 3:**
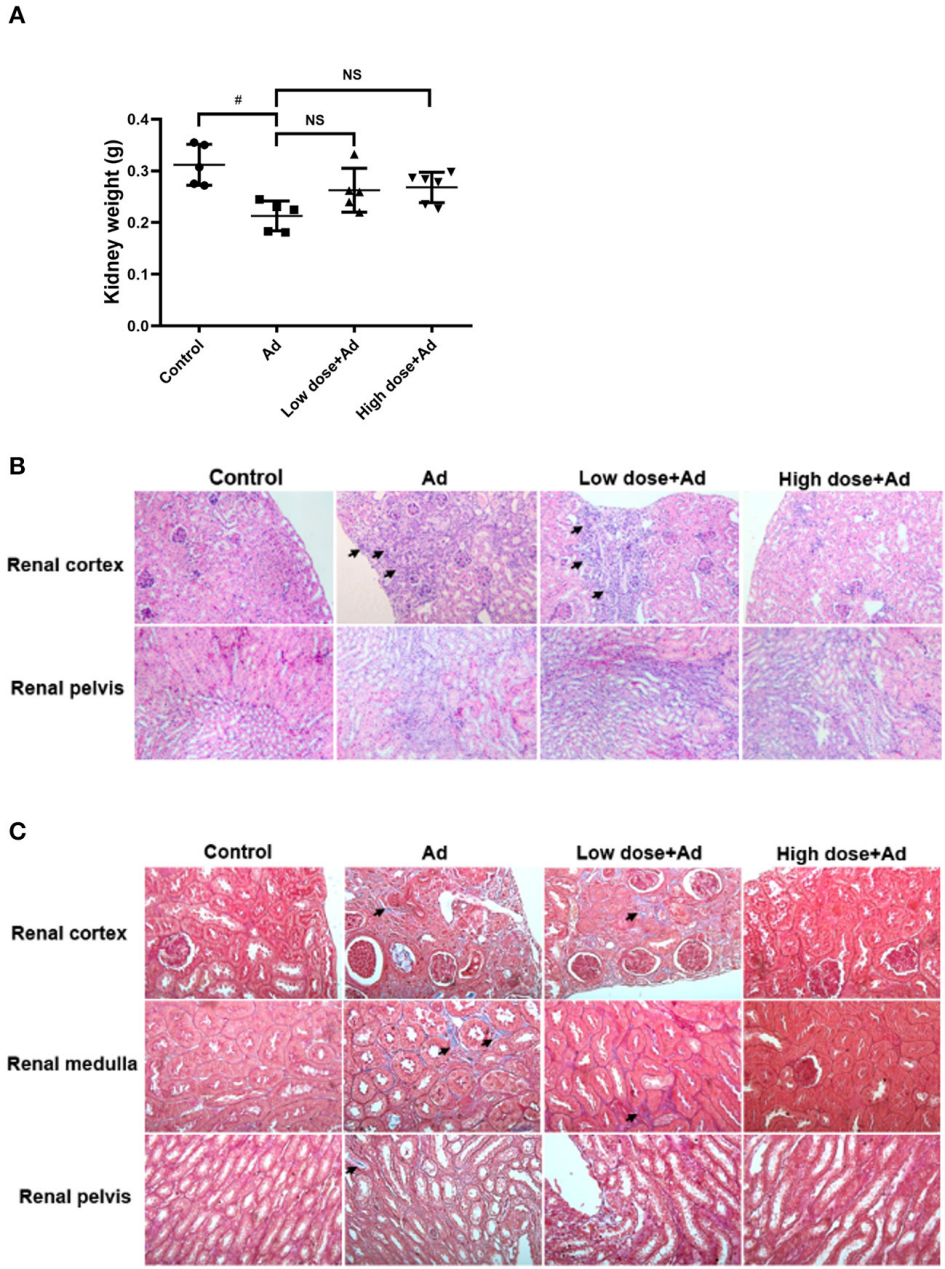
Gross examination and histological analysis of kidneys in mice with adenine (Ad)-induced chronic kidney disease (CKD), with or without probiotic administration. The kidneys of mice were collected and weighed. Histological sections of the renal cortex, renal medulla, and renal pelvis in Ad-induced CKD mice with or without probiotic treatment were studied. **(A)** Weights of the kidneys, **(B)** hematoxylin and eosin (H&E)-stained kidney sections, and **(C)** Masson's trichrome (MT)-stained kidney sections. The graphs represent mean ± SD obtained from five to six mice per group. Statistical analysis was performed by Kruskal–Wallis test (^#^*P* < 0.05; NS, not significant). The arrows in **(B)** and **(C)** indicate infiltration of inflammatory cells and tissue fibrosis, respectively. Mice without any treatment served as the experimental control (Magnification for H&E stain, 200× ; magnification for MT stain, 400×).

At the end of the study, Ad-mice showed a decrease in kidney weight when compared to controls. Probiotics treatment did not significantly increase kidney weight (Figure 3A). In the histopathological examination by H&E staining, there were a significantly increased infiltration of inflammatory cells, necrotic glomerular corpuscles in the renal cortex, and swelling of renal tubules in the renal pelvis of Ad-mice. After administration of probiotics in Ad-mice, there were fewer inflammatory cells in the renal cortex and glomerular corpuscles and normal compact renal tubules in the renal pelvis, particularly in Ad-mice treated with high-dose probiotics (Figure 3B). There was increased MT staining in the renal cortex, medulla, and pelvis of Ad-mice without treatment, whereas there was nearly no MT staining in controls or Ad-mice treated with high-dose probiotics (Figure 3C).

### Clinical Outcomes of the Human Study

**Figure 4:**
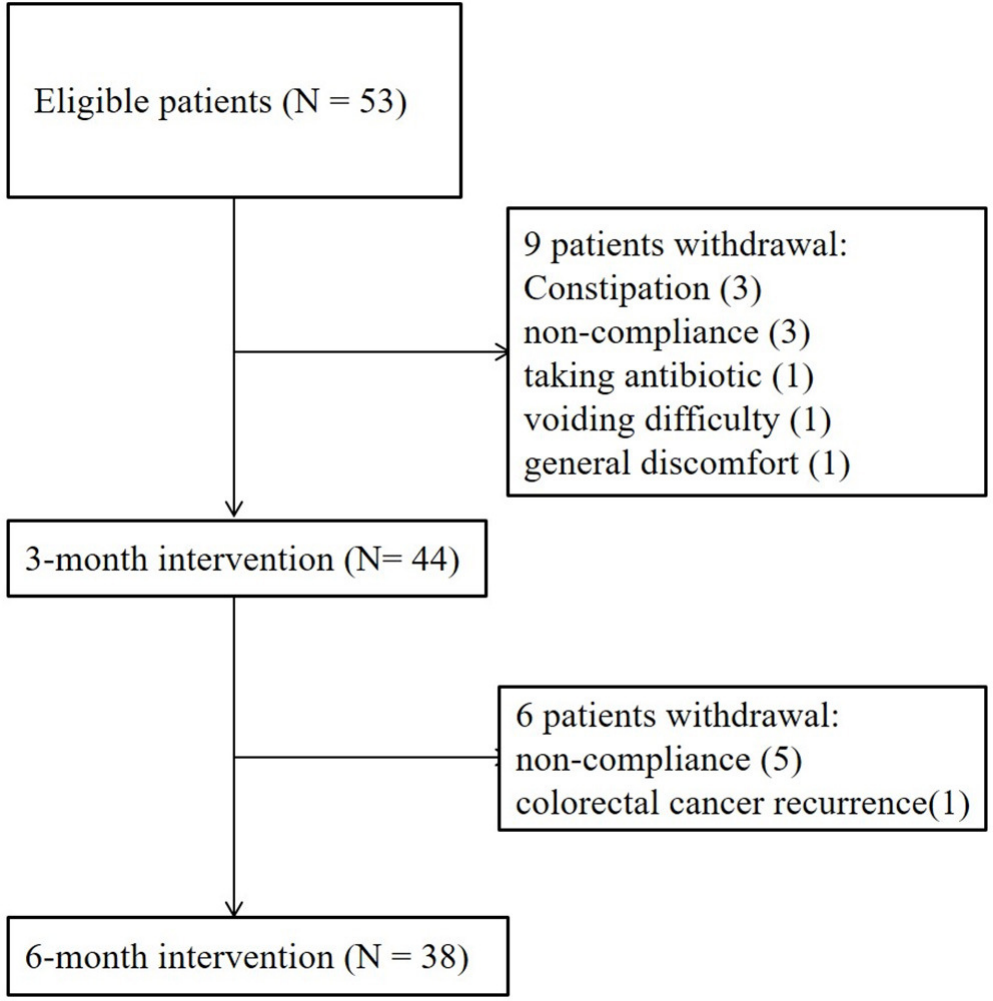
Clinical flowchart of patient enrollment.

A total of 53 patients were recruited for this study (Figure 4). A total of 15 patients withdrew because of non-compliance (8 patients), constipation (3 patients), voiding difficulty (1 patient), general discomfort (1 patient), taking antibiotics (1 patient), or cancer recurrence (1 patient). A total of 38 patients with CKD completed the 6-month study. There were no adverse events in any of the 38 patients. The main etiologies of CKD were chronic glomerulonephritis and diabetes mellitus (Table 1). The baseline levels of creatinine and eGFR were 2.77 ± 1.73 mg/dL and 30.16 ± 16.52 ml/min/1.73 m^2^, respectively. There was no significant change in blood pressure levels (systolic: from 135.1 ± 19.0 to 136.5 ± 17.7 mmHg; diastolic: from 74.1 ± 10.9 to 74.4 ± 13.7 mmHg) or body weight (from 65.0 ± 13.4 to 65.3 ± 14.0 kg) after the 6-month treatment (data not shown). The rate of decline in eGFR was significantly retarded, from −0.54 (−0.18, −0.91) to 0.00 (0.48, −0.36) ml/min/1.73 m^2^/month (*P* = 0.001), after the 6-month treatment (Table 2).

**Table 1:** General characteristics of the participants.

Age (years)	68.7 ± 13.1
Sex (F/M)	12/26
CKD stage (3/4/5)	(18/10/10)
Etiology of CKD	
Chronic glomerulonephritis	15
Diabetes mellitus	12
Gout	6
ADPKD	1
Analgesia nephropathy	1
Cisplatin related	1
Hypertension	1
Urolithiasis	1
Comorbidity	
Hypertension	36
Cardiovascular disease	17
Diabetes mellitus	17
Body weight (kg)	65.0 ± 13.4
Systolic blood pressure (mmHg)	135.1 ± 19.0
Diastolic blood pressure (mmHg)	74.1 ± 10.9
Serum creatinine (mg/dl)	2.77 ± 1.73
eGFR (ml/min/1.73 m^2^)	30.16 ± 16.52
Endotoxin (EU/ml)	0.96 (0.60, 1.47)
IL-6 (pg/ml)	3.24 (2.12, 5.28)
IL-18 (pg/ml)	319.00 (196.00, 517.60)
TNF-α (pg/ml)	14.54 (9.74, 26.45)

*F, female; M, male; CKD, Chronic kidney disease; ADPKD, autosomal dominant polycystic kidney disease; eGFR, estimated glomerular filtration rate; IL-6, interleukin-6; IL-18, interleukin-18; TNF-α, tumor necrosis factor-α*.

**Table 2:** Effects of probiotics on the rate of decline of the estimated glomerular filtration rate.

**6 months prior to intervention**		**Baseline**		**6 months after intervention**		**Prior to intervention**	**After intervention**
**Serum creatinine (mg/dL)**	**eGFR (ml/min/1.73 m^**2**^)**	**Serum creatinine (mg/dL)**	**eGFR (ml/min/1.73 m^**2**^)**	**Serum creatinine (mg/dL)**	**eGFR (ml/min/1.73 m^**2**^)**	**eGFR decline rate (ml/min/month/1.73 m^**2**^)**	**eGFR decline rate (ml/min/month/1.73 m^**2**^)**
2.42 ± 1.41	33.61 ± 17.31	2.77 ± 1.73	30.16 ± 16.52	2.89 ± 2.07	30.92 ± 18.04	−0.54 (−0.18, −0.91)	0.00 (0.48, −0.36)^*^

*Values are shown as mean ± SD, or median (25th, 75th percentile)*.

* *P < 0.01 (significant difference between before and after probiotic intervention)*.

*eGFR, estimated glomerular filtration rate*.

The values of cytokines and endotoxin were transformed using the natural log. The serum endotoxin levels [Ln (endotoxin)] decreased from 0.00 (−0.52, 0.37) to −0.33 (−1.99, 0.37) (*P* = 0.032) after the 6-month intervention (Table 3). Ln (IL-6) [from 1.21 (0.88, 1.57) to 0.63 (0.15, 1.07) (*P* = 0.006)], Ln (IL-18) [from 5.88 (5.48, 6.26) to 4.66 (4.18, 5.39) (*P* = 0.000)], and Ln (TNF-α) [from 2.53 (2.24, 2.84) to 2.18 (1.64, 2.66) (*P* = 0.024)] also decreased after the 6-month intervention.

**Table 3:** Effects of probiotics on the serum levels of endotoxin and proinflammatory cytokines.

	**Baseline**	**3 months after intervention**	**6 months after intervention**
Ln (endotoxin) (EU/ml)	0.00 (−0.52, 0.37)	0.12 (−0.66, 0.97)	−0.33 (−1.99, 0.37)^*^
Ln (IL-6) (pg/ml)	1.21 (0.88, 1.57)	1.19 (0.69, 1.58)	0.63 (0.15, 1.07)^**^
Ln (IL-18) (pg/ml)	5.88 (5.48, 6.26)	5.92 (5.48, 6.15)	4.66 (4.18, 5.39)^***^
Ln (TNF-α) (pg/ml)	2.53 (2.24, 2.84)	2.33 (1.98, 2.60)	2.18 (1.64, 2.66)^*^

*Values are shown as median (25th, 75th percentile)*.

* *P < 0.05*,

** *P < 0.01*,

*** *P < 0.001 (significant difference between before and after probiotic intervention)*.

Hard or muddy-to-watery stools tended to change to normal stool forms with probiotic treatment. The scores for borborygmus [2.6 ± 0.7 to 2.9 ± 0.4 (*P* = 0.013)] and flatulence [2.6 ± 0.6 to 2.9 ± 0.3 (*P* = 0.005)] increased significantly after the 6-month intervention (Table 4).

**Table 4:** Effects of probiotics on stool form and gastrointestinal symptoms.

	**Before intervention**	**3 months after intervention**	**6 months after intervention**
Stool form	3.0 ± 0.9, 3 (3, 3)	2.9 ± 0.5, 3 (3, 3)	2.9 ± 0.5, 3 (3, 3)
Ease of defecation	1.9 ± 0.5, 2 (2, 2)	1.9 ± 0.4, 2 (2, 2)	2.1 ± 0.5, 2 (2, 2)
Upper abdominal pain	2.9 ± 0.3, 3 (3, 3)	3.0 ± 0.0, 3 (3, 3)	3.0 ± 0.2, 3 (3, 3)
Lower abdominal pain	2.9 ± 0.3, 3 (3, 3)	2.9 ± 0.4, 3 (3, 3)	3.0 ± 0.2, 3 (3, 3)
Borborygmus	2.6 ± 0.7, 3 (2, 3)	2.8 ± 0.5, 3 (3, 3)	2.9 ± 0.4, 3 (3, 3)^*^
Flatulence	2.6 ± 0.6, 3 (2, 3)	2.9 ± 0.3, 3 (3, 3)^**^	2.9 ± 0.3, 3 (3, 3)^**^

*Values are shown as mean ± SD, and median (25th, 75th percentile)*.

* *P < 0.05*,

** *P < 0.01 (significant difference between before and after probiotic intervention)*.

**Figure 5:**
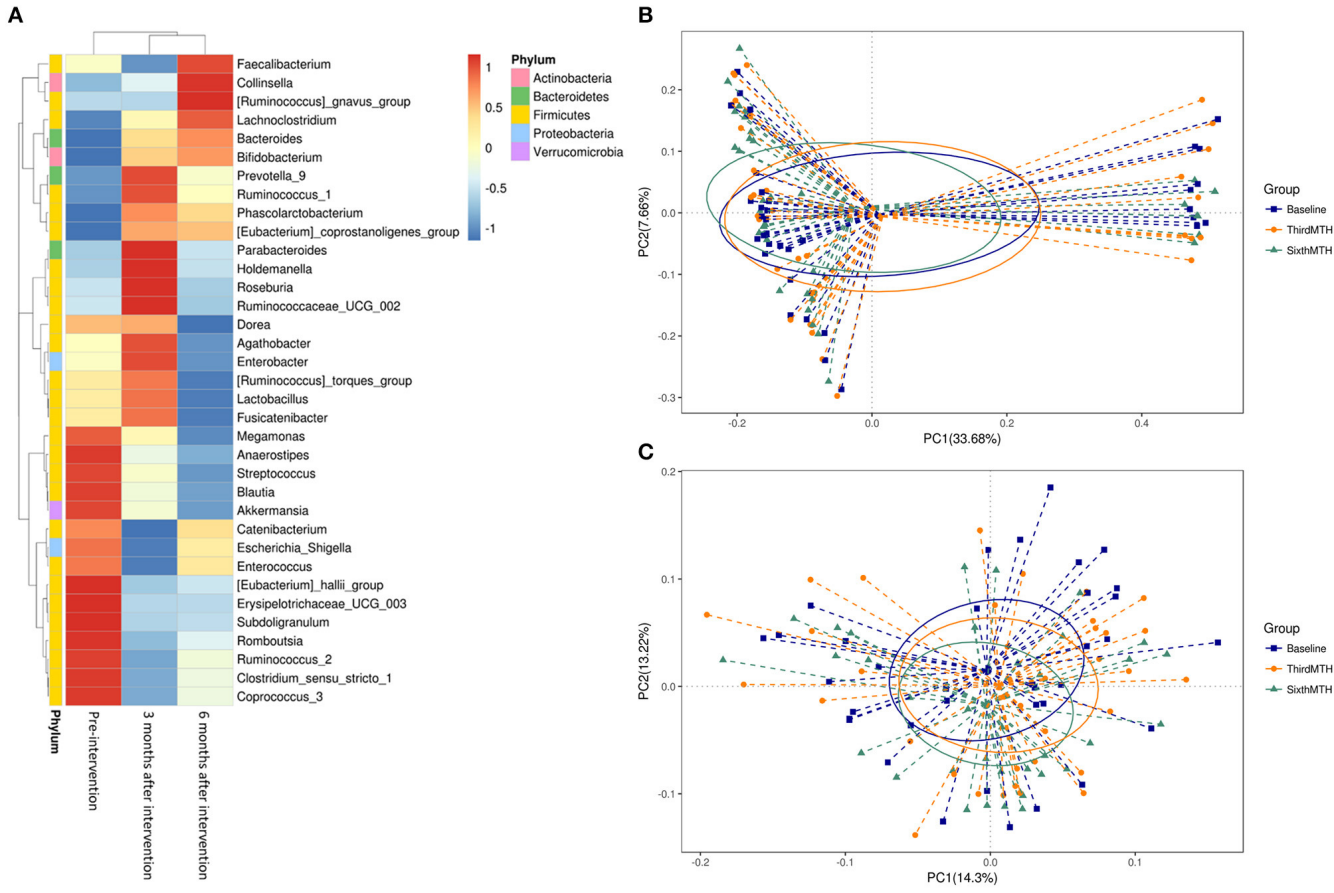
**(A)** Taxonomic composition of the genus level at pre-intervention and 3 and 6 months after intervention in the patients with CKD. Principal coordinates analysis (PCoA) plot. **(B)** unweighted UniFrac, **(C)** weighted UniFrac at pre-intervention and 3 and 6 months after intervention in the patients with CKD.

The NGS data for the gut microbiome in the patients with CKD revealed changes in the gut microbiota following probiotic administration (Figure 5). The red block represents the dominant gut microbiota in the genus. Before the intervention, the dominant genera of the intestinal microbiome were located at the blocks between *Megamonas* and *Coprococcus*_3. Three months after the probiotic intervention, the block between the *Prevotella*_9 and *Ruminococcaceae*_UCG_002 microbiota became the dominant genera in the gut. Six months after the probiotic intervention, the block between *Faecalibacterium* and *Bifidobacterium* became the dominant genera in the gut (Figure 5A). However, the scatter plot of unweighted and weighted Unifrac PCoA showed no significant changes in the gut microbiota after the probiotic intervention (Figures 5B,C), nor did other comparisons grouped by time, sex, age, and BMI reveal any difference.

**Figure 6:**
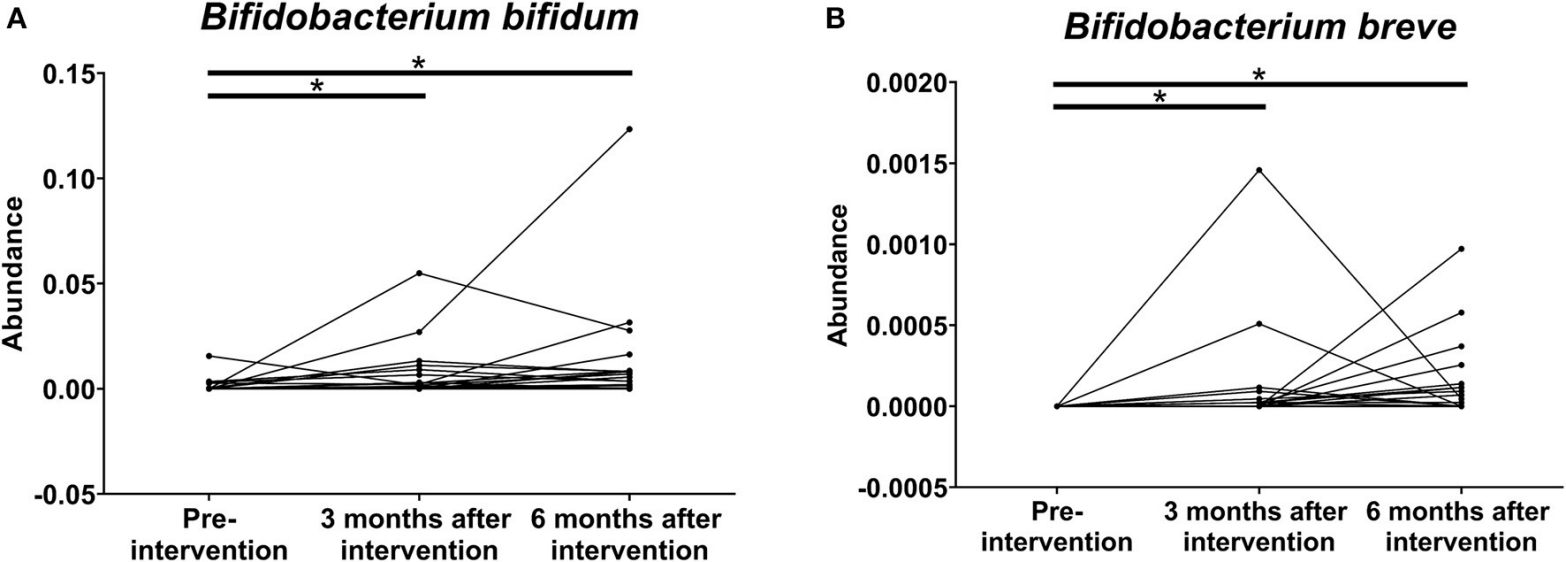
Effects of probiotics on the relative abundance of **(A)** *B. bifidum* and **(B)** *B. breve* on the stool microbiota at pre-intervention as well as 3 and 6 months after probiotic intervention in patients with chronic kidney disease (*P < 0.001).

The abundance of *B. bifidum* (Figure 6A) and *B. breve* (Figure 6B) in the stool microbiota also increased significantly after the 6-month intervention. The 16S rRNA gene amplicon sequencing analysis indicated that the abundance of *B. bifidum* in 4 patients stayed the same, that of *B. bifidum* in 29 patients increased, and that of *B. bifidum* in 3 patients decreased. On the other hand, the abundance of *B. breve* in 20 patients stayed the same, that of *B. breve* in 16 patients increased, and there were no patients with a decrease in the abundance of *B. breve*.

## Discussion

The present study demonstrated that probiotics could attenuate renal fibrosis and improve renal function in mice with CKD. In the human study, the deterioration of renal function was retarded after 6 months of probiotic intervention. In addition, serum levels of endotoxin, TNF-α, IL-6, and IL-18 decreased, and there was an improvement in borborygmus, flatulence, and stool form, as well as increased abundance of *B. bifidum* and *B. breve* in the stool microbiota.

Ranganathan et al. showed that the treatment with *Sporosarcina pasteurii* strain 6452 reduced BUN levels and significantly prolonged the life spans of 5/6 nephrectomy rats (22). Iwashita et al. reported that prebiotics (glutamine, dietary fiber, and oligosaccharide) and probiotics (*B. longum*) could ameliorate renal function deterioration in 5/6 nephrectomy rats (23). Yang et al. demonstrated that orally administered *L. rhamnosus* R0011 mitigated leaky gut and suppressed renal fibrosis in 5/6 nephrectomized mice (24). In addition, systemic inflammation was suppressed with decreased levels of serum TNF-α and IL-6 in the probiotic treatment group (24). In this study, the treatment with *L. acidophilus* (TYCA06), *B. longum* subspecies *infantis* (BLI-02), and *B. bifidium* (VDD088) could attenuate renal function deterioration and renal fibrosis in adenine-induced mice with CKD. In addition, the blood pressure of mice treated with probiotics was lower than that of untreated mice. In summary, *in vivo* experimental studies demonstrated the reno-protective effect of probiotics in the CKD animal models.

The results of clinical trials to date investigating probiotic administration in the patients with CKD have been inconsistent, and these studies did not enroll a sufficient number of participants. A randomized, double-blind, placebo-controlled crossover study reported that 3 months of treatment with 90 billion CFU a day of probiotics containing *L. acidophilus* KB27, *B. longum* KB25, and *S. thermophilus* KB19 significantly lowered the levels of BUN and improved quality of life in patients with stages 3 and 4 CKD (15). A randomized controlled trial showed that 6-week synbiotic therapy containing different strains across the genera *Lactobacilli, Bifidobacteria*, and *Streptococcus* did not significantly reduce serum indoxyl sulfate but did decrease serum p-cresyl sulfate in the patients with CKD (16). However, there were no significant changes in eGFR, serum inflammatory markers (IL-1β, IL-6, IL-10, and TNF-α), serum endotoxin concentration, or the Gastrointestinal Symptom Rating Scale (16). A small study involving nine hemodialysis patients showed that constipation improved after synbiotic treatment containing *L. casei* Shirota, *B. breve* Yakult, and galacto-oligosaccharides (18). A multi-center study revealed an improvement in gastrointestinal symptoms after 2 months of synbiotic treatment containing *L. acidophilus, B. lactis*, and inulin in 42 patients on hemodialysis (25). A randomized, double-blinded placebo-controlled study investigated the patients with PD receiving oral probiotics containing 10^9^ CFU *B. bifidum* A218, *B. catenulatum* A302, *B. longum* A101, and *L. plantarum* A87 per day (26). The serum levels of TNF-α, IL-5, and IL-6, and endotoxin decreased, and the serum levels of IL-10 increased after the 6-month treatment (26). In addition, residual renal function was preserved (26). In a 2-month small-scale randomized, double-blinded, placebo-controlled study from Mexico, symbiotic treatment increased *Bifidobacterium* counts in fecal samples among the patients on hemodialysis (27). In this study, renal function deterioration was attenuated after 6 months of probiotic combination intervention. In addition, there was a reduction in serum levels of endotoxin, TNF-α, IL-6, and IL-18; an improvement in stool form, borborygmus, and flatulence; and increased abundance of *B. bifidum* and *B. breve* in the stool microbiota. It is interesting to find that *B. breve*, which was not included in the multi-strain product formula, was also increased in the stools of the study subjects. This increase in beneficial bacteria of *Bifidobacterium* in the gut microbiota after probiotic treatment was also shown in our previous clinical trial on patients with atopic dermatitis treated with probiotics (28). Whether the other two strains in the same probiotics formula were colonized in the guts, the authors are not able to confirm from gut microbiota analysis using stools of the patients, which may need mucosal swab sampling for NGS analyses during colonoscopy examination.

The strength of this study was that it included both animal and human subjects, and the authors used NGS analysis to understand the effect of a probiotic combination on the gut microbiome in the patients with CKD. Our study results indicated that probiotic combinations might be used as an adjuvant therapy for patients with CKD. Moreover, changes in the gut microbiome evoked by probiotic combinations could also be regarded as assessment tools or biomarker signatures for the response of patients with CKD. However, the human study was limited by a small number of patients and a single-arm design. The lack of a placebo in the human study was a major limitation. A randomized, double-blinded, placebo-controlled design would have been better for proving efficacy.

## Conclusions

Probiotics could attenuate renal fibrosis and improve renal function in mice with CKD. In addition, probiotics could significantly retard the progression of renal failure; reduce the serum levels of endotoxin and proinflammatory cytokines (TNF-α, IL-6, and IL-18); improve stool form, borborygmus, and flatulence; and increase the abundance of *B. bifidum* and *B. breve* in the stool microbiota for the patients with CKD. Further large-scale, randomized, controlled trials are necessary to investigate the potential benefits of probiotics in the patients with CKD.

## Data Availability Statement

The datasets presented in this study can be found in online repositories. The name of the repository and accession number can be found at: National Center for Biotechnology Information (NCBI) BioProject, https://www.ncbi.nlm.nih.gov/bioproject/, PRJNA699722. The other raw data supporting the conclusions of this article will be made available by the authors, without undue reservation, to any qualified researcher.

## Ethics Statement

The animal study was reviewed and approved by the Animal Ethics Committee and the Institutional Animal Care and Use Committee (IACUC No. 106162), College of Medicine, National Cheng Kung University, Tainan, Taiwan. The studies involving human participants were reviewed and approved by Institutional Review Board and registered in clinical trials (NCT03228563). CKD patients were recruited at China Medical University Hospital, Taichung, Taiwan. The patients/participants provided their written informed consent to participate in this study.

## Author Contributions

I-KW, T-HY, P-SH, H-HH, C-YL, H-CL, and J-YW: conceptualization. I-KW: data curation, methodology, and writing/original draft preparation. Y-YH and Y-LK: formal analysis. Y-WK and Y-YH: investigation and validation. P-SH, H-HH, Y-WK, Y-YH, and Y-LK: visualization. I-KW and J-YW: writing/reviewing and editing. All authors contributed to the article and approved the submitted version.

## Conflict of Interest

P-SH, H-HH, Y-WK, and Y-YH were employed by the company Glac Biotech Co., Ltd., Tainan, Taiwan, and Y-LK was employed by the company Biotools Co., Ltd., New Taipei City, Taiwan. The remaining authors declare that the research was conducted in the absence of any commercial or financial relationships that could be construed as a potential conflict of interest.
